# Genomic Epidemiology of C2/H30Rx and C1-M27 Subclades of *Escherichia coli* ST131 Isolates from Clinical Blood Samples in Hungary

**DOI:** 10.3390/antibiotics13040363

**Published:** 2024-04-16

**Authors:** Kinga Tóth, Ivelina Damjanova, Levente Laczkó, Lilla Buzgó, Virág Lesinszki, Erika Ungvári, Laura Jánvári, Adrienn Hanczvikkel, Ákos Tóth, Dóra Szabó

**Affiliations:** 1 Institute of Medical Microbiology, Faculty of Medicine, Semmelweis University, 1089 Budapest, Hungary; 2 Department of Bacteriology, Parasitology and Mycology, National Center for Public Health and Pharmacy, 1097 Budapest, Hungarybuzgo.lilla@med.unideb.hu (L.B.); janvari.laura@nngyk.gov.hu (L.J.); toth.akos@nngyk.gov.hu (Á.T.); 3 One Health Institute, Faculty of Health Sciences, University of Debrecen, 4032 Debrecen, Hungary; 4 HUN-REN-DE Conservation Biology Research Group, University of Debrecen, 4032 Debrecen, Hungary; 5 HUN-REN-SE Human Microbiota Research Group, 1052 Budapest, Hungary; 6 Neurosurgical and Neurointervention Clinic, Semmelweis University, 1083 Budapest, Hungary

**Keywords:** whole genome sequencing (WGS), ST131, *Escherichia coli*, C1-M27, C2/H30RX, *bla*_CTX-M-27_, *bla*_CTX-M-15_, long-read sequencing

## Abstract

Extended-spectrum β-lactamase-producing *Escherichia coli* ST131 has become widespread worldwide. This study aims to characterize the virulome, resistome, and population structure of *E. coli* ST131 isolates from clinical blood samples in Hungary. A total of 30 C2/H30Rx and 33 C1-M27 ST131 isolates were selected for Illumina MiSeq sequencing and 30 isolates for MinION sequencing, followed by hybrid de novo assembly. Five C2/H30Rx and one C1-M27 cluster were identified. C1-M27 isolates harbored the F1:A2:B20 plasmid in 93.9% of cases. Long-read sequencing revealed that *bla*_CTX-M-27_ was on plasmids. Among the C2/H30Rx isolates, only six isolates carried the C2-associated F2:A1:B- plasmid type. Of 19 hybrid-assembled C2/H30Rx genomes, the *bla*_CTX-M-15_ gene was located on plasmid only in one isolate, while in the other isolates, IS*Ecp*1 or IS*26*-mediated chromosomal integration of *bla*_CTX-M-15_ was detected in unique variations. In one isolate a part of F2:A1:B- plasmid integrated into the chromosome. These results suggest that CTX-M-15-producing C2/H30Rx and CTX-M-27-producing C1-M27 subclades may have emerged and spread in different ways in Hungary. While *bla*_CTX-M-27_ was carried mainly on the C1/H30R-associated F1:A2:B20 plasmid, the IncF-like plasmids of C2/H30Rx or its composite transposons have been incorporated into the chromosome through convergent evolutionary processes.

## 1. Introduction

The dissemination of 3rd generation cephalosporin-resistant (3GCR) *Escherichia coli* (*E. coli*) strains has been driven by a few specific clones that have rapidly emerged in hospitals globally. The overuse and misuse of broad-spectrum antibiotics in healthcare institutions have led to the expansion of clonal variants showing resistance to three or more antibiotic classes (termed multidrug-resistant—MDR) [1]. These MDR clones are capable of causing severe infections, typically associated with limited treatment options, high morbidity, and mortality [2]. The highest disease burden in Europe is attributed to 3GCR *E. coli* [3], of which one of the most prominent clones is a group of *E. coli* strains with sequence type 131 (ST131). The high-risk *E. coli* clone ST131 includes mostly extraintestinal pathogenic *E. coli* (ExPEC) strains, which mainly cause bloodstream infections and urinary tract infections [3]. The main lineages of the ST131 clone belong to clade C, associated with fluoroquinolone resistance and extended-spectrum β-lactamase (ESBL) production. Clade C comprises two subclades: C1 and C2. While the C2 isolates defined as C2/H30Rx carry mainly the ESBL gene *bla*_CTX-M-15_, the C1 isolates defined as C1/H30R carry *bla*_CTX-M-14_ or *bla*_CTX-M-27_ genes [4,5,6]. Recently, a new sublineage within the C1, termed C1-M27, carrying *bla*_CTX-M-27_, has emerged as a common cause of infection next to the C2/H30Rx isolates [7]. The selective advantage of the spread of some ExPEC clones, such as ST131, was the acquisition of epidemic plasmids that allow the spread of CTX-M-type ESBLs and other antibiotic resistance genes within and even between species through horizontal gene transfer. Meanwhile, the plasmids have mostly co-evolved with bacterial genomes, allowing their hosts to spread clonally. In most cases, typical IncF-type plasmids co-evolved with the C1 and C2 subclades of ST131 [4,8,9]. Strains belonging to the C2/H30Rx subclade often carry the F2:A1:B- plasmid, while those belonging to the C1-M27 subclade carry the F1:A2:B20 plasmid carrying ESBL genes [4,8,10].

The results of a recent prospective cohort study showed that 24% (6/25) of invasive *E. coli* isolated from blood cultures at a major tertiary-care hospital in Budapest, Hungary, collected between October and November 2018, proved to be ESBL-producers. Whole genome sequence analysis showed that five *E. coli* isolates belonged to the ST131 clone. The study highlighted the dominance of the ST131, but due to the small sample size and short time period, it was unable to determine the population structure of the clone in Hungary [11].

This study aims to characterize *E. coli* ST131 isolates belonging to the C2/H30Rx or C1-M27 subclades collected from blood culture with spatiotemporal distribution from two time periods (2015–2018 and 2021) from Hungary through comparison and characterization of their virulome, resistome, major resistance plasmids and determination of their population structure. These two time periods were used to investigate possible changes in the population structure of ESBL-producing, invasive *E. coli* ST131 before and during the COVID-19 pandemic in Hungary.

## 2. Results

### 2.1. Selection of Isolates for the Study

Between 2015 and 2018 and in 2021, 59.6% (130/218) and 67.7% (157/232) of invasive ESBL-producing *E. coli* isolates investigated at the National Center for Public Health and Pharmacy (NCPHP) belonged to the ST131 clone where the ratio of C2/H30Rx and C1-M27 was 1 to 0.8 and 1 to 0.5, respectively. Based on the inclusion criteria, a total of 30 C2/H30Rx and 33 C1-M27 ESBL-producing *E. coli* ST131 isolates originated from 21 healthcare institutions in Hungary were selected for short read sequencing and 30 isolates (19 C2/H30Rx and 11 C1-M27) were selected for the long-read sequencing and for further analysis.

### 2.2. Antimicrobial Susceptibility

All isolates were found to be resistant to ceftriaxone and ciprofloxacin but susceptible to ceftazidime/avibactam, ertapenem, tigecycline, fosfomycin, imipenem, and meropenem. The C2/H30Rx isolates showed significantly lower susceptibility rates to ceftazidime, amikacin, tobramycin, and gentamicin than the C1-M27 ones. Two C1-M27 isolates showed resistance against colistin (Table 1).

### 2.3. Molecular Epidemiology

**Figure 1:**
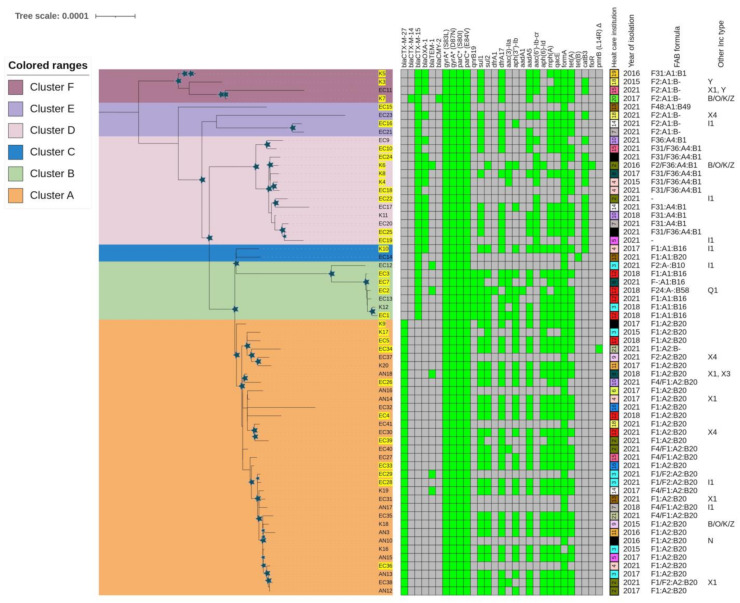
Maximum likelihood phylogeny of 63 ESBL-producing *E. coli* ST131 isolates and their genetic characteristic reconstructed by IQtree. Legend: Rectangles of different colors indicate clusters in the phylogenetic tree, and the star symbol indicates bootstrap values (LRT: >0.8 and UF bootstrap > 0.95). Hybrid genome assembly is indicated by a yellow background. In the table, the cells indicate the absence (grey) or presence of certain antibiotic-resistance genes (green). The symbol Δ indicates the mutation in chromosomally mediated colistin resistance, * the mutations in quinolone resistance determining region. The features show the profile of C1-M27 and C2/H30Rx isolates by health care institution, year of isolation, FAB formula, and other Inc types.

The phylogenetic tree supported the separation (i.e., aLRT > 0.8 and ultrafast bootstrap > 0.95) of the 33 C1-M27 isolates from all C2/H30Rx isolates (Figure 1). In the latter group, five clusters could be identified with similarly high statistical support (Figure 1 and Figure S2).

A total of 63 virulence factors and 29 antibiotic-resistance genes were detected in the two subclades. The median number of virulence genes was 25 (range 22 to 29), and of antibiotic resistance genes was 15 (range 6 to 16) among C1-M27 isolates versus 29 (range 24 to 31) and 14 (range 6 to 18) among C2/H30Rx isolates. (Figure 1 and Figure S1).

All C1-M27 isolates harbored *bla*_CTX-M-27_ and showed virotype C. The *traT* serum-resistance gene, *sen*B, *mcb*A, *pic* toxin genes, and some antibiotic resistance genes (*bla*_CTX-M-27_, *sul2*, *aph(3″)-Ib*, *aph(6)-Id*, *mph(A))* were significantly more frequent in the C1-M27 isolates than in the C2/H30Rx isolates (*p* < 0.05). All C2/H30Rx isolates harbored *bla*_CTX-M-15_. Out of 30 isolates, six exhibited virotype A, one virotype B, and 23 virotype C. Some adhesins (*afa* operon, *nfa*E, *pap* operon), toxins (*cnf1*, *hly* operon, *aslA*), invasins (*daaA-F*, *draA-D*), and the *hra* protectin gene, as well as some resistance genes (*bla*_CTX-M-15_, *bla*_OXA-1_, *aac(6′)-Ib-cr*, *qnrB19*, *catB3*), were present only in C2/H30Rx isolates (*p* < 0.01). The *aac(3)*-*IIa* occurred with higher frequency among C2/H30Rx subclade isolates (26.7% vs. 6.1%, *p* < 0.05).

Regarding the whole collection of isolates tested, an association was found between resistance to gentamicin and the presence of *aac(3)-IIa* (*p* < 0.001), between resistance to amikacin and the presence of *aac(6′)-Ib-cr* (*p* < 0.001), and between resistance to tobramycin and the presence of one of *aac(3)-IIa* or *aac(6′)-Ib-cr* (*p* < 0.001).

#### 2.3.1. Phylogenetic Analysis of *E. coli* ST131 Isolates

Within the C1-M27 subclade, one large cluster with 33 isolates could be identified, referred to as Cluster A. Cluster A isolates were collected from 18 healthcare institutions: three isolates from 2015, two from 2016, eight from 2017, four from 2018, and 16 isolates from 2021. The main plasmid replicon type was IncF (*n* = 33). Thirty-one out of 33 isolates had F1:A2:B20, one had F2:A2:B20, and another isolate had the F1:A2:B- FAB formula. The isolates carried other plasmid groups such as IncI (*n* = 2), IncX1 (*n* = 4), IncX3 (*n* = 1), IncX4 (*n* = 2), IncN (*n* = 1), and IncB/O/K/Z plasmid (*n* = 1). Col-like plasmid replicons were also present among Cluster A isolates: Col(MG828) (*n* = 29), Col156 (*n* = 30), Col(BS512) (*n* = 15), ColRNAI (*n* = 8), Col8282 (4), Col(pHAD28) (*n* = 2), and Col(KPHS6) (*n* = 1).

Within the C2/H30Rx subclade, five clusters could be identified (Cluster B–F). The isolates were collected from 15 healthcare institutions: two isolates from 2015, two from 2016, three from 2017, five from 2018, and 18 isolates from 2021. The main plasmid replicon type was IncF (*n* = 28), and the following FAB formulas were found: F31/F36:A4:B1 (*n* = 10), F2:A1:B- (*n* = 6), F1:A1:B16 (*n* = 5), F31:A1:B1 (*n* = 1), F-:A1:B16 (*n* = 1), F1:A1:B20 (*n* = 1), F2/F36:A4:B1 (*n* = 1), F2:A-:B10 (*n* = 1), F24:A-:B1 (*n* = 1), and F48:A1:B49 (*n* = 1), while two isolates did not carry IncF-type plasmid. Other Inc-types were found among the C2/H30Rx isolates, such as IncI (*n* = 5), IncX1 (*n* = 1), IncX4 (*n* = 1), IncB/O/K/Z plasmid (*n* = 1), IncY (*n* = 1), and IncQ1 (*n* = 1). Col-like plasmid replicons were also present in the following subclades: Col(MG828) (*n* = 9), Col156 (*n* = 16), Col(BS512) (*n* = 2), ColRNAI (*n* = 8), Col8282 (*n* = 6), and Col(pHAD28) (*n* = 8).

Seven isolates formed Cluster B. The isolates were collected in 2018 (n = 4 isolates) and 2021 (*n* = 3) from three healthcare institutions. Compared to the characteristic resistance and virulence genes of the C2 subclade, isolates of this cluster additionally carried *qnrB19* (*n* = 6), *sul2* (*n* = 5), *aph(6)-Id* (*n* = 5), and *aph(3″)-Ib* (*n* = 5) genes. Except for EC12, all isolates carried *astA* and *east1* virulence genes. All isolates exhibited virotype C except EC2, which had virotype B and additionally carried *iroBCDEN* genes. Five out of seven isolates carried plasmid with the F1:A1:B16 FAB formula.

Two isolates, which formed Cluster C, originated from different years and healthcare institutions and also possessed different FAB formulas (F1:A1:B20 and F1:A1:B16). Both isolates exhibited virotype C.

The thirteen isolates forming Cluster D were collected from nine different healthcare institutions and different years: one from each year except in 2021, when nine isolates were investigated. Compared to the characteristic resistance and virulence genes of the C2 subclade, isolates of this cluster additionally carried *hyl* operon and *cnf*1 genes. They showed virotype C and eleven isolates also carried *bla*_OXA-1_ and *aac(6′)-Ib-cr*. Ten of the 13 isolates possessed plasmid with the F31/F36:A4:B1 FAB formula.

Four isolates formed Cluster E, all collected in 2021 from four different healthcare institutions. In addition to the characteristic virulome and resistome for C2/H30Rx, EC15 and EC23 were identified by the presence of *nfaE*, *daaA-F*, *afa* operon, and *draA-F* virulence genes, the lack of the *pap* operon and exhibited virotype A. EC16 and EC21 exhibited virotype C. Three of the four isolates carried plasmid with the F2:A1:B- FAB formula.

The four isolates forming Cluster F were obtained from various years and different healthcare institutions. Each isolate harbored *nfaE*, *daaA-F*, and *afa* virulence genes, lacked *pap* operon, and exhibited virotype A. Three out of four isolates possessed the F2:A1:B- FAB formula.

#### 2.3.2. Localization and Genetic Environment of *bla*_CTX-M-15_ in the C2/H30Rx Isolates (Group A–I)

**Figure 2:**
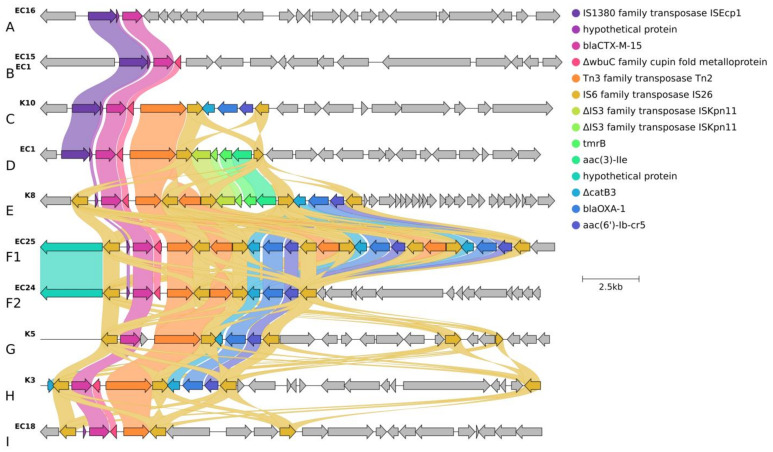
Linear sequence comparison of the genetic context (range 22,117 to 23,084 bp) of *bla*_CTX-M-15_ on chromosome and plasmid. Legend: Genes are grouped and color-coded according to function. The color-coded arrows indicated genes correspond to the circles in the top right corner of the figure. Grey arrows indicate genes with no similarity. Colorful wavy links represent the sequence identity of homologous gene groups identified by Clinker. Groups are indicated as “A–I” next to the designated isolate name and genome sequence.

The localization and genetic environment of *bla*_CTX-M-15_ genes were investigated in detail through 19 selected hybrid assembled genomes of the 30 C2/H30Rx isolates. Only one isolate from 2015 carried the *bla*_CTX-M-15_ located on a plasmid (F2:A1:B-). In the other 18 isolates, chromosomal integration of the *bla*_CTX-M-15_ linked to IS*Ecp1* (*n* = 7) or IS*26* (*n* = 12) translocable elements were detected. The group profile A-I was classified based on the structure of the genetic environment of each *bla*_CTX-M-15_ copy and its association with the upstream IS element (Figure 2).

In Group A, the IS*Ecp1*-linked orf-*bla*_CTX-M-15_ gene was present, and no other antibiotic-resistance genes were detected in its environment.

In Group B, the genetic environment of *bla*_CTX-M-15_ was similar to Group A, but it was followed by the 273 bp sequence of the gene encoding the cupin fold metalloprotein *wbu*C.

The genetic context observed in Group C consists of two distinct segments. The first segment corresponded to the Group B sequence (IS*Ecp1*-orf-*bla*_CTX-M-15_-*wbuC),* followed by a second segment. The second segment was a Tn*2* and IS*26*-linked composite transposon carrying Δ*catB3*, *bla*_oxa-1_ and *aac(6′)-Ib-cr5* resistance genes.

In Group D, the genetic environment of *bla*_CTX-M-15_ consisted of two different segments of transposable units. The first segment was identical to the Group B sequence. The second segment was an IS*26*-linked composite transposon carrying ΔIS*Kpn11*, *tmrB*, and *aac(3)-IIa.* The two segments were linked by Tn*2*.

In Group E, the IS*26*-linked composite transposon carrying orf-*bla*_CTX-M-15_-*wbuC* genes were present in the first segment and followed by two other different transposable unit segments. The second segment corresponded to the second segment of Group D (ΔIS*Kpn11*-*tmrB*-*aac(3)-IIa*), and the third segment corresponded to the second segment of Group C (Δ*catB3*-*bla*_oxa-1_-*aac(6′)-Ib-cr5*).

In Group F, the genetic environment of *bla*_CTX-M-15_ genes consisted of two different segments of transposable units. The first segment corresponded to the IS*26*-linked *bla*_CTX-M-15_. The IS*26*-linked composite transposon carried Δ*catB3*-*bla*_oxa-1_-*aac(6′)-Ib-cr5* was the second segment. The two segments were linked by Tn*2*. Group F2 had one repeat, while Group F1 had three repeats of the second segment.

In Group G, the genetic environment of *bla*_CTX-M-15_ genes consisted of two segments. The first segment harbored IS*26*-linked *bla*_CTX-M-15_ like in Group F but lacked the orf gen between IS*26* and *bla*_CTX-M-15_, and the second segment resembled the second segment of Group F2.

In Group H, the genetic environment of *bla*_CTX-M-15_ was very similar in structure to Group G, but both segments were localized on plasmid.

In Group I, the IS*26*-linked orf- *bla*_CTX-M-15_–*wbuC-*Tn*2*-IS*26* was present, and no other antibiotic resistance genes were detected in its environment.

For IS*Ecp1*-linked *bla*_CTX-M-15_ (Group A–D), one copy of the Group A structure was found in one isolate (EC16–Cluster E), three copies of the Group B structure were found in one isolate (EC15–Cluster E), where one of the IS*Ecp1*-mediated insertion sequences was located in an opposite orientation. One copy of the Group C structure was observed in the K10 isolate (Cluster C). All four isolates in Cluster B (EC1, EC2, EC3, EC7) carried 2 copies of the IS*Ecp1*-linked *bla*_CTX-M-15_ gene inserted into identical positions in their bacterial chromosome. One copy of *bla*_CTX-M-15_ belonged to the Group B structure, while the second copy belonged to the Group D structure, which was located 84,842 bp downstream.

For IS26-linked *bla*_CTX-M-15_ (Group E–I), one copy of the Group E structure was found in four isolates (K4, K6, K8, EC19–Cluster D), one copy of the Group F1 in one isolate EC25 (Cluster F), and one copy of the Group F2 in two (EC24 and EC22) isolates.

The K5 isolate formed Group G, the F31/F36:A1:B1-like plasmid integrated into the chromosome, where the IS26 mediated translocable unit was inserted upstream next to the 23S ribosomal RNA coding region.

The Group H structure was located on an F2:A1:B-like plasmid (91,036 bp), which was uniquely found in the K3 isolate (Cluster F). The Group I structure was observed in three isolates (EC10, EC18–Cluster D, and K7–Cluster F).

#### 2.3.3. Localization and Genetic Environment of *bla*_CTX-M-27_ in the C1-M27 Isolates (Region I–III)

**Figure 3:**
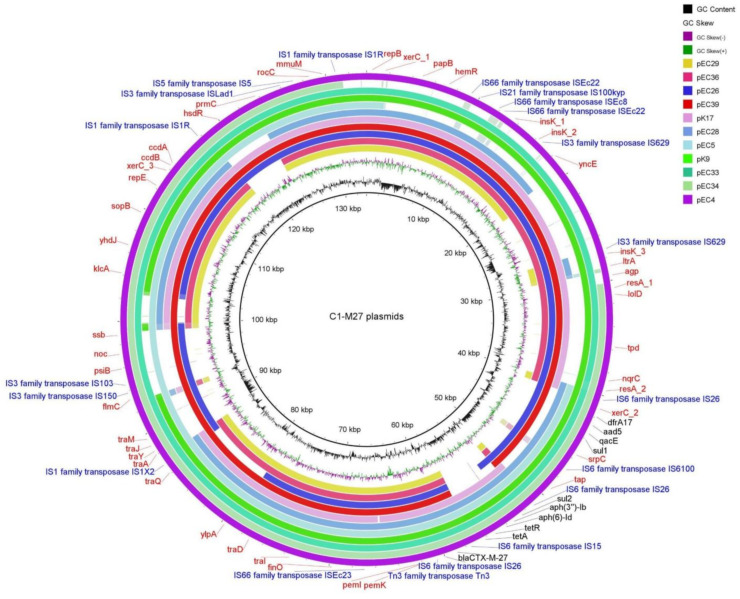
BRIG representation of eleven IncF plasmids carrying *bla*_CTX-M-27_ in ESBL-producing *Escherichia coli* ST131 C1-M27 isolates. Legend: The comparisons are made in reference to pEC4. The inner rings show GC content (black) and GC skew (purple/green). The remaining rings show BLAST comparisons of eleven IncF plasmids carrying *bla*_CTX-M-27_ of C1-M27 *E. coli*. The outer ring highlights the genes of pEC4, shown in different colors. The genomic features of annotated genes are indicated and color-coded in red. AMR genes are indicated in black and MGE in blue.

All investigated *bla*_CTX-M-27_ genes were located on IncF-like plasmids (Figure 3). The assembled plasmids were 133,550 bp for EC4, 98,865 bp for EC5, 134,637 bp for EC26, 96,681 bp for EC28, 80,412 bp for EC29, 137,256 bp for EC33, 105,606 bp for EC34, 100,608 bp for EC36, 127,181 bp for EC39, 139,893 bp for K9, and 110,045 bp for K17 in size. Nine out of eleven investigated isolates carried IncF(F1:A2:B20) plasmid, one carried IncF(F2:A2:B20), and another isolate had IncF(F1:A2:B-).

**Figure 4:**
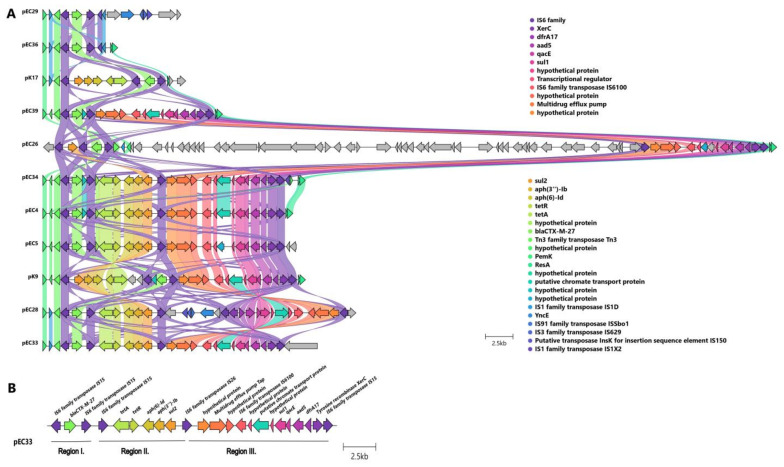
Linear sequence comparison of the genetic environment of *bla*_CTX-M-27_. Legend: Genes are grouped and color-coded according to the function of the genes. The color-coded arrows indicated genes correspond to the circles in the top right corner of the figure. Grey arrows indicate genes with no similarity. Colorful wavy links represent the sequence identity of homologous gene groups. (**A**) Genetic composition of 11 plasmids containing *bla*_CTX-M-27_. (**B**) Genetic environment of EC3 with Regions. Regions are indicated R on the figure. Three similar regions of resistance genes were present in the plasmid genetic context. Region I. consists of *bla*_CTX-M-27_ and Region II. consists of *tetA*, *aph(6)-Id*, *aph(3″)-Ib*, sul2. Region III. consists of *dfrA*17, *aadA*5, *qacEΔ1*, *sul1*, and *mph(A)*.

The antibiotic resistance genes were encoded in three regions of the IncF plasmids (Figure 4, Table 2): Region I. with *bla*_CTX-M-27_ (10/11 was carried by *IS6* family-linked composite transposon); Region II. with *te*t*A*, *aph(6)-Id*, *aph(3″)-Ib*, *sul2* (six/seven was carried by IS*6* family-linked composite transposon); Region III. with *dfrA17*, *aadA5*, *qacEΔ1*, *sul1*, *mph(A)* (six/eight was carried by IS*6* family-linked composite transposon). EC29 and EC36 isolates carried Region I. alone, and K17 isolates possessed Regions I. and II. and EC26 and EC39 isolates possessed Regions I. and III. Six isolates (EC4, EC5, EC28, EC33, EC34, and K9) possessed Regions I., II., and III.

## 3. Discussion

This genomic epidemiology study revealed for the first time the population structure of the C1-M27 and C2/H30Rx *E. coli* ST131 clones isolated from blood cultures in Hungary. This study was based on our previous observation study [11], which highlighted the dominance of the ST131 clone among ESBL-producing *E. coli* strains isolated from blood samples.

In Hungary, a CTX-M-15-producing ST131 clone was first identified in 2010, and later, the first ESBL-producing invasive *E. coli* isolates belonging to the C1-M27 subclade were detected in 2015. Since then, the number and rate of ESBL-producing ST131 clones and their subclades have gradually increased until 2018 [11]. In 2015–2018 and in 2021, a similar proportion of invasive ESBL-producing *E. coli* isolates investigated at the National Center for Public Health and Pharmacy belonged to the ST131.

In this study, all isolates proved to be resistant to ceftriaxone and ciprofloxacin, but the CTX-M-15-producing isolates showed higher resistance rates than the CTX-M-27-producing isolates to ceftazidime. Although CTX-M-27 also has the Asp240Gly amino acid substitution that is responsible for ceftazidime resistance in CTX-M-15, generally lower MIC values can be measured in vitro [12,13,14]. All isolates were susceptible to carbapenems. This finding is supported by data from EARS-Net, where only one carbapenem-resistant isolate was reported from Hungary between 2015 and 2021. The two colistin-resistant *E. coli* C1-M27 isolates (EC34 and EC35) (3.2% resistance rate in this collection) originated from the same healthcare institution in 2021. Similar low levels of colistin resistance were observed in a previous report from Hungary, where out of 146 investigated *E.coli* isolates, one isolate showed *mcr*-1 related colistin-resistance, and six isolates were colistin-tolerant in 2010–2011, and four isolates were colistin-tolerant in 2016 [15]. In this study, none of the isolates possessed any plasmid-mediated colistin resistance (*mcr*) genes. Only the EC34 had *pmrB* (L14R) mutation, which is strongly associated with colistin resistance [16]. Each of the 63 isolates possessed the same amino acid substitution in *pmrB* (E123D), which has been described in the context of chromosome-mediated colistin resistance [17,18]. However, they remained colistin-susceptible (except for two isolates). A Korean study also found *E. coli* isolates with *pmrB* (E123D) substitution as colistin-susceptible [19]. Thus, these results are still ambiguous and the origin of the resistance was not identified in one isolate and requires further investigation. The C2/H30Rx isolates showed lower susceptibility rates than the C1-M27 ones to amikacin, tobramycin, and gentamicin due to aminoglycoside-modifying enzymes [20,21]. Among the aminoglycoside-modifying enzymes, the *aac(6′)-Ib-cr* was present only in C2/H30Rx isolates, while *aac(3)-IIa* and *aph(6)-Id* were present both in C2/H30Rx and C1-M27 isolates. According to the scientific literature, *aac(3)-IIa* confers resistance to gentamicin and tobramycin, while *aac(6′)-Ib-cr* to amikacin and tobramycin [22]. In the study, the same associations were found between the presence of genes of these aminoglycoside-modifying enzymes and corresponding antibiotics. The identified *gyrA* and *parC* mutations are well known to confer resistance to ciprofloxacin. Additional resistance mechanisms such as *qnrA* or *aac(6′)-Ib-cr* may also contribute to fluoroquinolone resistance. Among the isolates of the C2 subclade, *qnrA* and *aac(6′)-Ib-cr* were present [23,24].

It has been shown that IncFII-type plasmids are mainly associated with the *bla*_CTX-M-15_ gene [4]. In this study, among the 19 C2/H30Rx isolates, only one isolate from 2015 carried the C2-associated F2:A1:B- plasmid harboring *bla*_CTX-M-15_. The remaining 18 isolates showed chromosomal integration of the *bla*_CTX-M-15_ gene in one or several copies. The chromosomal integration of *bla*_CTX-M-15_ has been described before in a few studies that reported a local distribution [25,26]. However, these 19 isolates were delivered from five clusters and sixteen healthcare institutions. These data indicate that C2/H30Rx ST131 clusters harboring chromosomal *bla*_CTX-M-15_ might have emerged convergently and spread across Hungary.

There was a difference in the case of the type of IS, which was responsible for the translocation of the genetic environment of the *bla*_CTX-M-15_. Two types of IS-mediated translocable elements were detected: IS*Ecp1* was located upstream of the *bla*_CTX-M-15_ gene or IS*26* was incorporated upstream and downstream of the *bla*_CTX-M-15_. There were also IS*26* or IS*Ecp1* transposon structures that consisted of overlapping IS*26*-based translocatable units. The IS*26* is one of the few IS types that have been shown to form fusions between two DNA sequences via replication and form cointegrates rather than move alone to a new location [27,28]. IS*26* could also be found in arrays, intercalated next to other transposable elements, and could form units able to undergo tandem amplification in drug-resistant plasmids or chromosomes [29]. Like IS*26*, the IS*Ecp1*-mediated transposition proceeds via one IS*Ecp1*, mediating genetic transposition events that involve homologous recombination. Both IS families could be responsible for the higher level of expression of the *bla*_CTX-M_ genes, which play an important role in the dissemination of antibiotic-resistance genes among Gram-negative bacteria [30]. The phenomenon of IS*Ecp1* and/or IS*26*-mediated *bla*_CTX-M-15_ transposition has been described before in a few studies [31,32,33,34,35]. Shawa et al. described the co-occurrence of IS*Ecp1*- *bla*_CTX-M-15_ and other *catB3*, *bla*_oxa-1_, *aac(6′)-Ib-cr5* genes on the chromosomal environment of CTX-M-15-producing ST131 *E. coli* [31,36].

In this study, the chromosomal integration of one or more copies of *bla*_CTX-M-15_ and its genetic environment was mediated either by IS*Ecp1* or by IS*26.* Additionally, the IS26-mediated *bla*_CTX-M-15_ gene was followed by three copies of Tn*2*-IS*26*-Δ*catB3*-*bl*a_OXA-1_-*aac(6′)-Ib-cr5*-IS2*6* segment in one isolate (Group F1).

Shropshire et al. described IS*26*-mediated amplification of *bla*_CTX-M-15_ and *bla*_OXA-1_ in the carbapenem-resistant ST131 *E. coli* genome and hypothesized that the IS*26*-*bla_CTX-M-15_*-ΔTn*2* could drive the amplification of the genetic environment of *bla*_CTX-M-15_ [34,37]. In this study, amplification mediated by IS*Ecp1* also occurred in four isolates.

The results suggest that the IncFII plasmid may have been progressively integrated into chromosomes in the 2010s and later was progressively lost in the genetic environment of *bla*_CTX-M-15_. The Cluster B isolates with two copies of *bla*_CTX-M-15_ probably emerged independently in Hungary.

Therefore, apart from the horizontal gene transfer of the plasmids encoding *bla*_CTX-M-15_, the composite transposon-linked antimicrobial resistance genes had undergone several chromosomal insertion events mediated by IS*26* or IS*Ecp1*. The independent convergent appearance of various IS-mediated chromosomal integration of antimicrobial resistance genes suggests that this process may have an evolutionary potential. The benefit of the chromosomal integration of the *bla*_CTX-M-15_ and other AMR genes might contribute to maintenance and dissemination under the selection pressure of the antimicrobial environment. The plasmid may impose a fitness burden on their hosts but also provides diversity, promoting the worldwide dissemination of successful clones [38,39,40].

Unlike the C2 subclade, in C1-M27 isolates all *bla*_CTX-M-27_ and AMR genes were located on IncF plasmids. The majority of all C1-M27 ST131 isolates carried an F1:A2:B20 plasmid. The F1:A2:B20 plasmid type is strongly associated with the C1/H30R subclade [6]. Here, all the IS*6* family-linked composite transposons carrying AMR genes remained on the plasmid, in line with many examples in the literature [41,42]. Similar Regions (I., II., III.) consisting of AMR genes had been found before in other F1:A2:B20 plasmids of C1-M27 isolates, and AMR gene regions without *bla*_CTX-M-27_ were found in F1:A2:B20 plasmid with *bla*_CTX-M-14_ [9,43]. In this collection, one isolate with F1:A2:B- plasmid harbored the same Region I., II., III. while there were F1:A2:B20 isolates harboring Region I. or I. and III.

In the study, the most common Col-like replicon type in the C1 and C2 subclades was Col156 (30 vs. 16), followed by Col(MG828) (29 vs. 9), respectively. The exact role of Col-like plasmids or replicons is not clear. Col-like plasmids or replicons are mobilizable vectors that have been described as promoting the spread of antibiotic resistance plasmids via horizontal gene transfer in the *Enterobacteriaceae* family [36,44,45].

## 4. Materials and Methods

### 4.1. Bacterial Collection

The putative ESBL-producing *E. coli* isolates obtained from blood samples have been submitted to the NCPHP for confirmation and molecular typing from the whole country. All isolates were identified using matrix-assisted laser desorption ionization time-of-flight mass spectrometry (MALDI Biotyper, Bruker, Bremen, Germany). The Double Disc Synergy Test (DDST) was used for confirmation of ESBL production: the DDST confirmation test was performed using the “ESβL Detection Disc Set” (MAST Diagnostica GmbH, Reinfeld, Germany) according to the manufacturer’s instruction [46,47]. The clonal relationship was examined using *XbaI*-pulsed-field gel electrophoresis (PFGE) in all ESBL-producing *E. coli* isolates [48]. For the determination of ST131 subclades, the PFGE results and multiplex PCR were used as described by Matsumura [49].

Non-duplicate isolates were selected for further investigation based on the PFGE results and covering spatiotemporal distribution during 2015–2018 and 2021 (Supplementary Table S1).

### 4.2. Antimicrobial Susceptibility Testing

The antibiotic susceptibility testing of all isolates was performed using the disc diffusion method. Where the disc diffusion method was not recommended (e.g., colistin) or where MIC values gave additional significance to the disc diffusion results, MIC values were determined. The antimicrobial susceptibility testing to ceftriaxone, cefotaxime, fosfomycin, ceftazidime/avibactam, ertapenem, ciprofloxacin, imipenem, meropenem, gentamicin, amikacin, tobramycin, and tigecycline was performed by disk diffusion (Mast Diagnostica GmbH, Reinfeld, Germany), to ceftriaxone, cefotaxime, fosfomycin, ceftazidime/avibactam, ertapenem by MIC Test Strips (Liofilchem, Roseto degli Abruzzi, Italy), to colistin by MICRONAUT MIC-Strip (MERLIN Diagnostika GmbH, Bornheim, Germany) and interpreted using EUCAST guidelines [50]. ATCC 25,922 *E. coli* reference strain was used for quality control of antimicrobial susceptibility testing.

### 4.3. Molecular Characterization

#### 4.3.1. DNA Extraction

The Bacterial DNA was extracted and purified with the DNeasy UltraClean Microbial Kit (Qiagen, Hilden, Germany) according to the manufacturer’s instructions. The assessment of DNA quality was carried out using a TapeStation 2200 automated electrophoresis system (Agilent Technologies, Palo Alto, CA, USA) and DNA quantity was measured using a Qubit 3.0 Fluorometer (Invitrogen, Life Technologies, Carlsbad, CA, USA).

#### 4.3.2. Short-Read Sequencing

Library preparation for short-read sequencing was performed with an Illumina DNA Prep kit (Illumina, San Diego, CA, USA). Whole genome sequencing of the isolates was performed on an Illumina MiSeq platform (150-bp paired-end sequencing). Raw data were processed by default using the EnteroBase pipeline [51]. EnteroBase provides automated pipelines that implement multiple functions that allow users to upload their own sequencing data for de novo assembly using a streamlined pipeline. Once the reads are received, EnteroBase provides the following workflow: First, automatic assembly with QAssembly. QAssembly consists of quality assemblies, including read pre-processing, trimming (QAssembly: Sickle), assembly (QAssembly: Spades), post-correction and filtering (QAssembly: BWA), and converting results to fasta format (QAtoFasta). This is followed by a quality assessment of the assemblies (QA evaluation). The fasta files were retrieved and analyzed using additional bioinformatics tools [51].

The quality of draft genome assemblies was considered appropriate for downstream analyses if the average sequencing depth of contigs exceeded 50-fold, their total size matched the expected genome size (5 ± 0.3 Mb), and N50 was longer than 100 kbp. Detailed genomic quality indicators are available in Supplementary Table S2.

#### 4.3.3. Long-Read Sequencing and Genome Assembly

After short-read sequencing of the isolates, a phylogenetic tree was constructed. The antimicrobial resistance genes, virulence genes, mobile genetic elements, and plasmid replicon types were identified within the clusters. Based on these results, isolates were selected for long-read sequencing to represent the main characteristics and clusters proportionally, with their corresponding isolation years (2015–2018 and 2021) and healthcare institutions.

The long-read sequencing library of the selected isolates was prepared by using the Ligation Sequencing Kit (SQK-LSK109, Oxford Nanopore, Oxford, UK) according to the manufacturer’s instruction for genomic DNA with barcoding (Native Barcoding Expansions 96 (EXP-NBD196, Oxford Nanopore, Oxford, UK) and the sequencing was performed on MinION Mk1C (Oxford Nanopore, Oxford, UK). Basecalling (with fast basecalling mode) and demultiplexing of the long-read raw sequences were performed with Guppy v5.1.12 as implemented in MinKNOW v 21.11.6. Raw long-read data processing was performed using the following GalaxyEU (https://nanopore.usegalaxy.eu) (accessed on 4 January 2023) toolkit with default parameters: Porechope v0.2.4 [52] was used for quality trimming and removing barcodes. We carried out multiple assemblies: first, we assembled the long-reads using Flye v2.9.1 [53] nd polished the assemblies, then used a short-read first hybrid assembly approach as implemented in Unicycler v0.5.0 [54] with default parameters. BWA MEM v0.7.17 [55] was used for short-read alignment, and Pilon v1.20 [56] was used for polishing. To decrease the mismatch rate, another round of genome polishing was applied. Polished Flye and polished Unicycler assemblies were analyzed simultaneously. Finally, those assemblies with less fragmentation were selected as representative genome assemblies of the isolates: 22 Flye assemblies and eight Unicycler assemblies were obtained for further analysis. Assembly statistics were retrieved with Quast v5.0.2 [57]. Prokka v1.14.6 was used for the genome annotation of the assemblies [58].

Clinker v0.0.27 [59] was used to compare and visualize gene cluster homology (genes were predicted using Prokka v1.14.6). The plasmid content and mobile genetic elements (MGE) were analyzed by PlasmidFinder v2.1 [60], pMLST v2.0 [60] and Nucleotide BLAST (https://blast.ncbi.nlm.nih.gov/Blast.cgi) (accessed on 9 January 2023) and MGE v1.0.3 [61] online tools. The sequence similarity of plasmid sequences was analyzed using BRIG v0.95 [62].

#### 4.3.4. Phylogenomic Reconstruction and Clustering of Isolates

The genome annotation prepared with Prokka was used as input for Panaroo v1.1.2 [63] to reconstruct the core genome alignment of the isolates. The default values of Panaroo were used, except that the frequency threshold of genes for inclusion in the core alignments was increased to 99% (--core-threshold 0.99). A total of 3788 genes were included in the core genome. Phylogenetic relationships of the isolates were reconstructed using IQtree v2.2.3 [64], with automatic model selection with ModelFinderPlus turned on (-m MFP), and the robustness of the results was assessed using 1000 aLRT and 1000 ultrafast bootstrap replications. The fastBAPS v1.0.8 [65] R v4.2.2 [66] package was used to identify clusters in the population structure using optimise_baps priors. Instead of using a single threshold, the fastBAPS algorithm was applied that relies on statistical genetic models to effectively partition molecular variation [67] and is independent of the phylogenetic reconstruction. The “optimise.baps” BAPS priors were optimized with the optimise_prior function. Then, the fastp_baps and best_baps_partition functions implemented in the fastbaps R package were used to obtain the best clustering scheme describing the genetic clusters in the dataset. Both IQtree and fastBAPS used the multiple sequence alignment of the complete core genome as input. The results of fastBAPS clustering with the phylogenetic tree were visualized using the R package ggtree v3.6.2 [68], for which the phylogenetic tree was midpoint rooted using phangorn v2.11.1 [69]. The phylogenetic trees were visualized and annotated with sample metadata using the Interactive Tree of Life (iTOL v6.7.4) web tool [70].

#### 4.3.5. Virulence and Antibiotic Resistance Genes Analysis

The antimicrobial resistance genes (AMR) in the complete genome sequences were identified using ResFinder v4.1 [71,72,73] CGE online tools (https://www.genomicepidemiology.org/) (accessed on 7 January 2023).

Virulence factor genes were retrieved using VirulenceFinder v2.0 [74] and Virulence Factors Database (VFDB v2020-Feb-28 through SeqSphere+ [75]) online tools. The virotypes A to D of the ST131 isolates were assigned according to the scheme developed by Blanco et al., 2013 [76].

### 4.4. Statistical Analysis

For statistical analysis, Fisher’s exact test was performed in an online program (https://www.socscistatistics.com/tests/fisher/default2.aspx) (accessed on 15 March 2023). Three approaches were used for statistical tests: 1. testing for independence between two variables (presence or absence of virulence or resistance gene compared to two subclade isolates); 2. testing for independence between two variables (presence or absence of phenotypic antibiotic resistance compared to two subclade isolates); 3. testing for independence between two variables (phenotypic antibiotic resistance compared to presence or absence of antibiotic resistance genes). For each comparison, *p* < 0.05 was considered to be statistically significant.

## 5. Conclusions

These results indicate that C2 and C1 subclades may have emerged and spread in different ways in Hungary. The C1-M27 variant has formed one cluster since its appearance in Hungary around 2015–2016. This hypothesis is further supported by the fact that C1-M27 isolates showed high similarities in the virulome and resistome, and 93.9% of them harbored an F1:A2:B20 plasmid. In contrast, the C2/H30Rx clusters may have appeared independently in the country. In the meantime, the composite transposons or a part of the IncF-like plasmids may have been integrated into the chromosome and later progressively lost from the genetic environment of *bla*_CTX-M-15_. By the time the C1-M27 clone appeared, C2/H30Rx clones with chromosomally encoded *bla*_CTX-M-15_ were already present in Hungary. The C2 subclade could have undergone a convergent evolutionary process in Hungary, and these clonal lineages were still detectable in 2021. Although the composite transposons ensuring the presence of ESBL and other AMR genes in the C1 clonal lineage can be linked to *IS*26, as in C2, it has not been transferred into a chromosomal environment and underwent a clonal expansion different from C2. Also, neither C1 nor C2 subclades have been able to displace each other but have been able to stably coexist and spread. Within the C2 subclade, the Cluster B isolates with two copies of *bla*_CTX-M-15_ showed local clonal expansion and probably emerged independently in Hungary.

## Figures and Tables

**Table 1:** Antimicrobial susceptibility of the *E. coli ST131* isolates.

Antimicrobial Agent	C2/H30Rx			C1-M27			*p*-Value
	R% (*n* = 30)	MIC_50_ (*n* = 30)	MIC_90_ (*n* = 30)	R% (*n* = 33)	MIC_50_ (*n* = 33)	MIC_90_ (*n* = 33)	
FOS	0	2	4	0	2	4	1
ETP	0	0.0625	0.125	0	0.016	0.0625	1
CZA	0	0.25	0.5	0	0.125	0.25	1
CAZ	83.9	16	64	15.6	4	8	<0.001 *
CRO	100	256	256	100	128	256	1
COL	0	0.25	0.5	6.3	0.25	1	0.5
TGC	0	ND	ND	0	ND	ND	1
AK	40	ND	ND	6.1	ND	ND	<0.01 *
TM	66.7	ND	ND	3.0	ND	ND	<0.001 *
GM	40	ND	ND	0	ND	ND	<0.001 *
MEM	0	ND	ND	0	ND	ND	1
IMI	0	ND	ND	0	ND	ND	1
CIP	100	ND	ND	100	ND	ND	1

Legend: Comparison of the susceptibility of 33 C1-M27 and 30 C2/H30Rx ST131 *E. coli* to different antibacterial agents. The antibiotics tested were fosfomycin (FOS), ertapenem (ETP), ceftazidime/avibactam (CZA), ceftazidime (CAZ), ceftriaxone (CRO), colistin (COL), tigecycline (TGC), amikacin (AK), tobramycin (TM), gentamicin (GM), meropenem (MEM), imipenem (IMI), and ciprofloxacin (CIP). The R% corresponds to the resistance rate; MIC corresponds to the minimum inhibitory concentrations. MIC_50/90_ is the MIC value at which ≥50% and ≥90% of isolates are inhibited. ND refers to not conducted. * Statistic value was revealed using Fisher’s exact test: *p* < 0.05.

**Table 2:** Main genetic characteristics of ST131 *E. coli* isolates with hybrid assembled genomes.

Subclade	Cluster Type	Cluster-Specific Virulence Gene	Localization of *bla*_CTX-M_	IS Element Linked to *bla*_CTX-M_	Copy of *bla*_CTX-M_	Genetic Environment of *bla*_CTX-M_					Additional Antibiotic Resistance Genes	Corresponding Isolate (s)
						Categorization of the Genetic Environment of *bla*_CTX-M_	1st Segment	2nd Segment	3rd Segment	4th Segment		
C1-M27	A	*traT*, *senB*, *mcbA*, *pic*	plasmid	IS6 family	1	Regions I	*bla*_CTX-M-27_	-	-	-	-	EC29, EC36
						Regions I–II		*tetA*-*aph(6)-Id*-*aph(3″)-Ib*-*sul2*	-	-		K17
						Regions I–III		*dfrA17*- *aadA5*-*qacEΔ1*-*sul1*-*mph(A)*	-	-		EC39, EC26
						Regions I–II–III		*tetA*-*aph(6)-Id*-*aph(3″)-Ib*-*sul2*	*dfrA17- aadA5-qacEΔ1-sul1-mph(A)*	-		EC4, EC5, EC28, EC33, EC34, K9
C2/H30Rx	B	*astA*, *east1*	chromosomal	ISEcp1	2	Group B, D	*bla*_CTX-M-15_	*tmrB*-*aac(3)-Iia*	-	-	*qnrB19*, *sul2*, *aph(6)-Id*, *aph(3″)-Ib*	EC1, EC2, EC3, EC7
	C	-			1	Group C		*ΔcatB3*-*bla*_oxa-1_-*aac(6′)-Ib-cr5*	-	-	-	K10
	D	*hyl*, *cnf*1		IS26		Group I		-	-	-		EC10, EC18
						Group F2		*ΔcatB3*-*bla*_oxa-1_-*aac(6′)-Ib-cr5*	-	-		EC24, EC22
						Group F1		*ΔcatB3*-*bla*_oxa-1_-*aac(6′)-Ib-cr5*	*ΔcatB3*-*bla*_oxa-1_-*aac(6′)-Ib-cr5*	*ΔcatB3*-*bla*_oxa-1_-*aac(6′)-Ib-cr5*		EC25
						Group E		*tmrB*-aac(3)-Iia	*ΔcatB3-bla_oxa-1_-aac(6′)-Ib-cr5*	-		K4, K6, K8, EC19
	E	-		ISEcp1	3	Group B		-	-	-		EC15
		*nfaE*, *daaA-F*, *afa* operon, *draA-D*			1	Group A		-	-	-		EC16
	F			IS26		Group I		-	-	-		K7
			chromosomal			Group G		*ΔcatB3*-*bla*_oxa-1_-*aac(6′)-Ib-cr5*	-	-		K5
			plasmid			Group H		*ΔcatB3*-*bla*_oxa-1_-*aac(6′)-Ib-cr5*	-	-		K3

Legend: The symbol “-“ indicates the absence of a genetic element.

## Data Availability

The raw data of long-reads and short-reads are available on the Sequence Read Archive (SRA) database under the BioProject number PRJNA1003301.

## Supplementary Materials

The following supporting information can be downloaded at https://www.mdpi.com/article/10.3390/antibiotics13040363/s1. The data set supporting the results of this article is included within the article and in Supplementary Data Sheet 1: Table S1: Thirty C2/H30Rx and thirty-three C1-M27 ESBL-producing *E. coli* ST131 were used in the study, including the source, ST131 sublineage, collection date, sex and age; Table S2:The genomic quality indicators for sequencing of the 63 ESBL-producing *E. coli* ST131;Figure S1: Maximum likelihood phylogeny of sixty-three, ESBL-producing *E. coli* ST131 isolates and their genetic characteristic, including virulome and virotype; Figure S2.: The results of phylogenetic reconstruction and fastBAPS clustering using the core genome alignment prepared by Panaroo.
