# Supplementary material for: Genomic Epidemiology of C2/H30Rx and C1-M27 Subclades of Escherichia coli ST131 Isolates from Clinical Blood Samples in Hungary
Source: Antibiotics (Basel). 2024 Apr 16;13(4):363. doi: 10.3390/antibiotics13040363 (PMC11047377; doi:10.3390/antibiotics13040363)
Supplement: Supplementary file 1 [file antibiotics-13-00363-s001.zip › Supplementary Materials.pdf]

Supplementary Materials

The thirty isolates selected for MiniON sequencing are the followings:

C2/H30Rx: EC1, EC2, EC3, EC7, EC10, EC15, EC16, EC18, EC19, EC22, EC24, EC25, K3, K4, K5, K6, K7, K8, K10

C1-M27: EC4, EC5, EC26, EC28, EC29, EC33, EC34, EC36, EC38, EC39, K9

Table S1. Thirty C2/H30Rx and thirty-three C1-M27 ESBL-producing *E. coli* ST131 were used in the study, including the source, ST131 sublineage, collection date, sex and age.

Number	Isolate	Health care Institution	City of Isolation	ST131 Sublineage	Collection Date	Sex	Age
1	AN10	1	Esztergom	C1-M27	Aug 29, 2016	female	52
2	AN12	2	Pécs	C1-M27	Feb 28, 2017	male	54
3	AN13	3	Budapest	C1-M27	May 17, 2017	female	59
4	AN14	4	Kaposvár	C1-M27	Apr 23, 2017	male	78
5	AN15	5	Salgótarján	C1-M27	May 07, 2017	male	86
6	AN16	6	Szentes	C1-M27	Sep 19, 2017	female	75
7	AN17	7	Eger	C1-M27	Jun 24, 2018	male	87
8	AN18	8	Miskolc	C1-M27	Oct 26, 2018	female	91
9	AN3	11	Kecskemét	C1-M27	Apr 08, 2016	female	68
10	EC1	12	Budapest	C2/H30Rx	Oct 13, 2018	female	63
11	EC2	12	Budapest	C2/H30Rx	Oct 16, 2018	male	83
12	EC3	12	Budapest	C2/H30Rx	Oct 30, 2018	male	77
13	EC4	12	Budapest	C1-M27	Oct 12, 2018	male	38
14	EC5	12	Budapest	C1-M27	Oct 24, 2018	female	58
15	EC7	8	Miskolc	C2/H30Rx	Feb 25, 2021	female	67
16	EC9	10	Miskolc	C2/H30Rx	Oct 19, 2021	male	66
17	EC10	15	Debrecen	C2/H30Rx	Apr 11, 2021	female	46
18	EC11	15	Debrecen	C2/H30Rx	Oct 05, 2021	male	86
19	EC12	3	Budapest	C2/H30Rx	Jul 10, 2021	female	44
20	EC13	12	Budapest	C2/H30Rx	Jul 14, 2021	female	87
21	EC14	13	Budapest	C2/H30Rx	Mar 26, 2021	female	92

22	EC15	13	Budapest	C2/H30Rx	Nov 27, 2021	male	49
23	EC16	14	Kistarcsa	C2/H30Rx	Oct 03, 2021	male	78
24	EC17	14	Kistarcsa	C2/H30Rx	Dec 12, 2021	male	64
25	EC18	4	Kaposvár	C2/H30Rx	Jul 31, 2021	male	71
26	EC19	5	Salgótarján	C2/H30Rx	Jun 27, 2021	male	77
27	EC20	7	Eger	C2/H30Rx	Feb 06, 2021	male	75
28	EC21	7	Eger	C2/H30Rx	May 06, 2021	female	62
29	EC22	2	Pécs	C2/H30Rx	Apr 21, 2021	male	70
30	EC23	18	Sopron	C2/H30Rx	Sep 22, 2021	female	52
31	EC24	1	Esztergom	C2/H30Rx	Dec 09, 2021	male	72
32	EC25	1	Esztergom	C2/H30Rx	Sep 10, 2021	female	89
33	EC26	10	Miskolc	C1-M27	Oct 21, 2021	female	80
34	EC27	15	Debrecen	C1-M27	Jan 14, 2021	male	64
35	EC28	3	Budapest	C1-M27	Apr 10, 2021	female	95
36	EC29	3	Budapest	C1-M27	Oct 21, 2021	female	91
37	EC30	12	Budapest	C1-M27	Apr 20, 2021	male	74
38	EC31	13	Budapest	C1-M27	Jul 23, 2021	male	70
39	EC32	16	Kecskemét	C1-M27	Jul 07, 2021	male	76
40	EC33	16	Kecskemét	C1-M27	Jun 03, 2021	male	74
41	EC34	21	Nyíregyháza	C1-M27	Aug 15, 2021	male	64
42	EC35	21	Nyíregyháza	C1-M27	Jun 16, 2021	female	81
43	EC36	4	Kaposvár	C1-M27	May 01, 2021	male	20
44	EC37	9	Salgótarján	C1-M27	Mar 11, 2021	female	60
45	EC38	2	Pécs	C1-M27	Jan 07, 2021	female	72
46	EC39	2	Pécs	C1-M27	Apr 27, 2021	male	0
47	EC40	2	Pécs	C1-M27	Sep 09, 2021	male	80
48	EC41	18	Sopron	C1-M27	Aug 27, 2021	male	67
49	K3	18	Sopron	C2/H30Rx	Aug 23, 2015	male	85
50	K4	4	Kaposvár	C2/H30Rx	Dec 18, 2015	male	66
51	K5	19	Kazincbarcika	C2/H30Rx	Apr 19, 2016	male	50
52	K6	2	Pécs	C2/H30Rx	May 19, 2016	female	59
53	K7	20	Szolnok	C2/H30Rx	Feb 28, 2017	female	64
54	K8	8	Miskolc	C2/H30Rx	Feb 09, 2017	female	68
55	K9	1	Esztergom	C1-M27	Sep 18, 2017	male	78
56	K10	4	Kaposvár	C2/H30Rx	Dec 25, 2017	female	91
57	K11	10	Miskolc	C2/H30Rx	Jun 24, 2018	male	70

58	K12	3	Budapest	C2/H30Rx	Nov 05, 2018	male	86
59	K16	3	Budapest	C1-M27	Jul 07, 2015	male	82
60	K17	3	Budapest	C1-M27	Apr 18, 2015	male	74
61	K18	9	Vác	C1-M27	Sep 08, 2015	male	73
62	K19	14	Kistarcsa	C1-M27	Mar 24, 2017	male	58
63	K20	11	Kecskemét	C1-M27	Aug 16, 2017	male	72

Table S2. The genomic quality indicators for sequencing of the 63 ESBL-producing *E. coli* ST131

Isolate	Average Coverage (Assembled)	Length (Assembled)	N50	Contig Count (Assembled)	GC-Content (Assembled)	Assembly Base Count	Approximated Genome Size (Mbases)	Avg. Contig Length (Assembled)
AN10	52	5100310	192059	108	50.7	5102603	5.1	47246
AN12	162	5091666	241485	95	50.74	5092650	5.1	53606
AN13	158	5086337	222583	98	50.73	5088098	5.1	51919
AN14	103	5118088	222568	111	50.63	5121005	5.1	46135
AN15	79	5068428	222567	104	50.73	5070486	5.1	48754
AN16	93	5051891	222568	141	50.74	5054074	5.1	35844
AN17	224	5263647	174043	172	50.72	5269929	5.3	30639
AN18	76	5124846	209775	108	50.69	5127450	5.1	47476
AN3	97	5127402	230034	101	50.75	5129275	5.1	50784
EC1	102	5321807	209574	163	50.75	5325715	5.3	32673
EC2	98	5307214	217637	128	50.73	5309255	5.3	41478
EC3	77	5302430	217637	142	50.78	5303689	5.3	37349
EC4	104	5042280	334258	91	50.76	5044297	5.0	55431
EC5	110	5071469	209000	94	50.71	5072071	5.1	53958
EC7	96	5285885	209434	161	50.76	5289394	5.3	32853
EC9	111	5372281	178598	141	50.59	5374259	5.4	38115
EC10	104	5412460	188604	133	50.63	5415014	5.4	40714
EC11	119	5322663	205891	143	50.69	5324680	5.3	37235
EC12	118	5391808	208914	134	50.71	5394177	5.4	40255
EC13	111	5296905	190448	150	50.74	5299437	5.3	35329
EC14	117	5201436	185632	119	50.72	5203294	5.2	43725
EC15	93	5171236	375675	116	50.67	5173684	5.2	44600
EC16	100	5354612	244894	135	50.72	5357210	5.4	39683
EC17	92	5330428	222425	123	50.68	5332573	5.3	43354
EC18	129	5330645	211292	142	50.66	5333255	5.3	37558
EC19	122	5281740	227913	113	50.63	5285081	5.3	46770
EC20	117	5415385	191062	139	50.59	5418007	5.4	38978
EC21	136	5236652	208561	139	50.67	5238763	5.2	37688
EC22	106	5232131	178138	113	50.54	5234025	5.2	46318
EC23	112	5228388	303673	102	50.73	5230137	5.2	51275
EC24	108	5353169	183048	139	50.7	5356682	5.4	38537
EC25	128	5373045	187963	141	50.66	5375894	5.4	38126
EC26	107	5126382	209304	128	50.77	5129318	5.1	40072

EC27	113	5105730	217443	126	50.73	5108654	5.1	40544
EC28	123	5225969	222525	124	50.84	5229044	5.2	42169
EC29	157	5124527	222604	135	50.75	5127665	5.1	37982
EC30	138	5066166	209103	100	50.69	5068660	5.1	50686
EC31	120	5080630	227816	99	50.63	5082398	5.1	51337
EC32	109	5062971	216752	99	50.77	5065269	5.1	51164
EC33	66	5112246	209517	107	50.72	5115021	5.1	47803
EC34	124	5067023	139115	118	50.72	5069750	5.1	42963
EC35	131	5130156	222583	122	50.74	5132354	5.1	42068
EC36	136	5073026	216213	103	50.75	5074957	5.1	49271
EC37	156	5194576	191062	134	50.64	5196419	5.2	38779
EC38	103	5183088	222525	126	50.69	5185330	5.2	41153
EC39	155	5029882	222436	99	50.77	5033399	5.0	50842
EC40	154	5091402	216129	127	50.73	5095568	5.1	40122
EC41	132	5160021	249550	130	50.75	5164137	5.2	39724
K3	134	5253038	258335	156	50.74	5257504	5.3	33701
K4	116	5378258	222570	135	50.67	5379827	5.4	39850
K5	122	5167652	282373	135	50.75	5171665	5.2	38308
K6	105	5451821	170814	209	50.73	5457074	5.5	26110
K7	141	5342460	209516	158	50.84	5345802	5.3	33834
K8	100	5425918	188563	156	50.65	5428631	5.4	34798
K9	98	5175699	216213	102	50.63	5176766	5.2	50752
K10	132	5313776	172776	158	50.71	5316493	5.3	33648
K11	117	5412730	190448	160	50.59	5416664	5.4	33854
K12	117	5307291	192201	157	50.73	5310814	5.3	33826
K16	94	5109157	241468	86	50.75	5110155	5.1	59420
K17	97	5184632	209000	111	50.71	5186313	5.2	46723
K18	101	5007092	255223	89	50.74	5008246	5.0	56272
K19	96	5287332	216213	128	50.74	5288974	5.3	41320
K20	86	5104267	199234	120	50.74	5106747	5.1	42556

Figure S1. Maximum likelihood phylogeny of sixty-three, ESBL-producing *E. coli* ST131 isolates and their genetic characteristic, including virulome and virotype. **Legend:** Rectangles of different colors indicate clusters in the phylogenetic tree and the star symbol indicates bootstrap values (LRT: > 0.8 and UF bootstrap > 0.95). Hybrid genome assembly is indicated by yellow background. In the table, the cells indicate the absence (grey) or presence of certain virulence genes (red). The features show the profile of C1-M27 and C2/H30Rx isolates by year of isolation, health care institution and virotypes

Figure S2. The results of phylogenetic reconstruction and fastBAPS clustering using the core genome alignment prepared by Panaroo. **Legend:** The results of phylogenetic reconstruction and fastBAPS clustering using the core genome alignment prepared by Panaroo. The left panel shows the Maximum Likelihood (ML) phylogenetic tree reconstructed by IQtree and midpoint rooted with phangorn. Branch lengths on the phylogram are proportional to the pairwise genetic divergence of isolates. The right panel shows the clusters of isolates identified using fastBAPS. All columns represent different clusters and the fields in blue in the different columns indicate the classification of the isolate in the corresponding cluster.
