# Supplementary figures and images for: Genomic Epidemiology of C2/H30Rx and C1-M27 Subclades of Escherichia coli ST131 Isolates from Clinical Blood Samples in Hungary
Source: Antibiotics (Basel). 2024 Apr 16;13(4):363. doi: 10.3390/antibiotics13040363 (PMC11047377; doi:10.3390/antibiotics13040363)

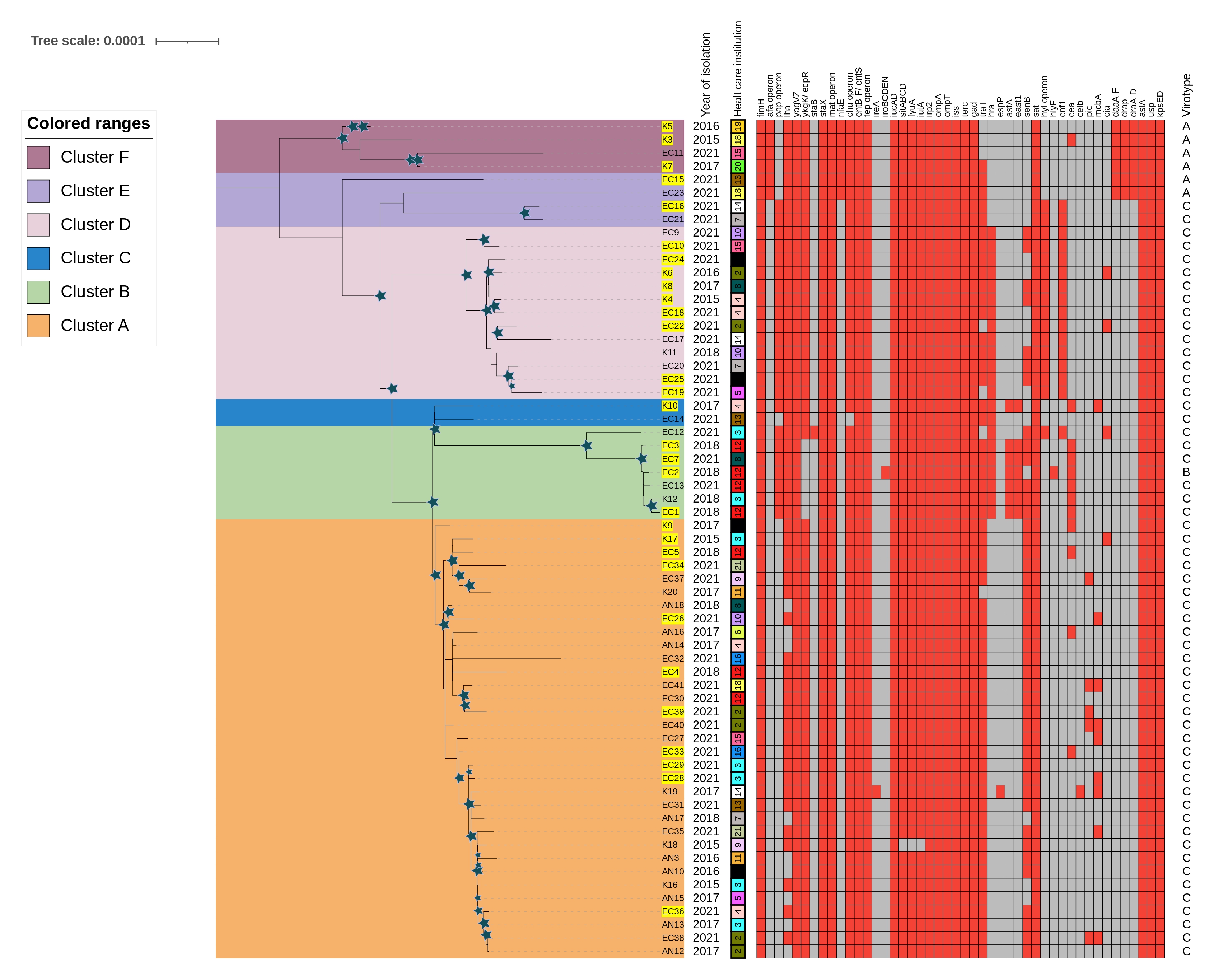

Supplement: Supplementary file 1 [file antibiotics-13-00363-s001.zip › Supplementary Figure S1.JPEG]

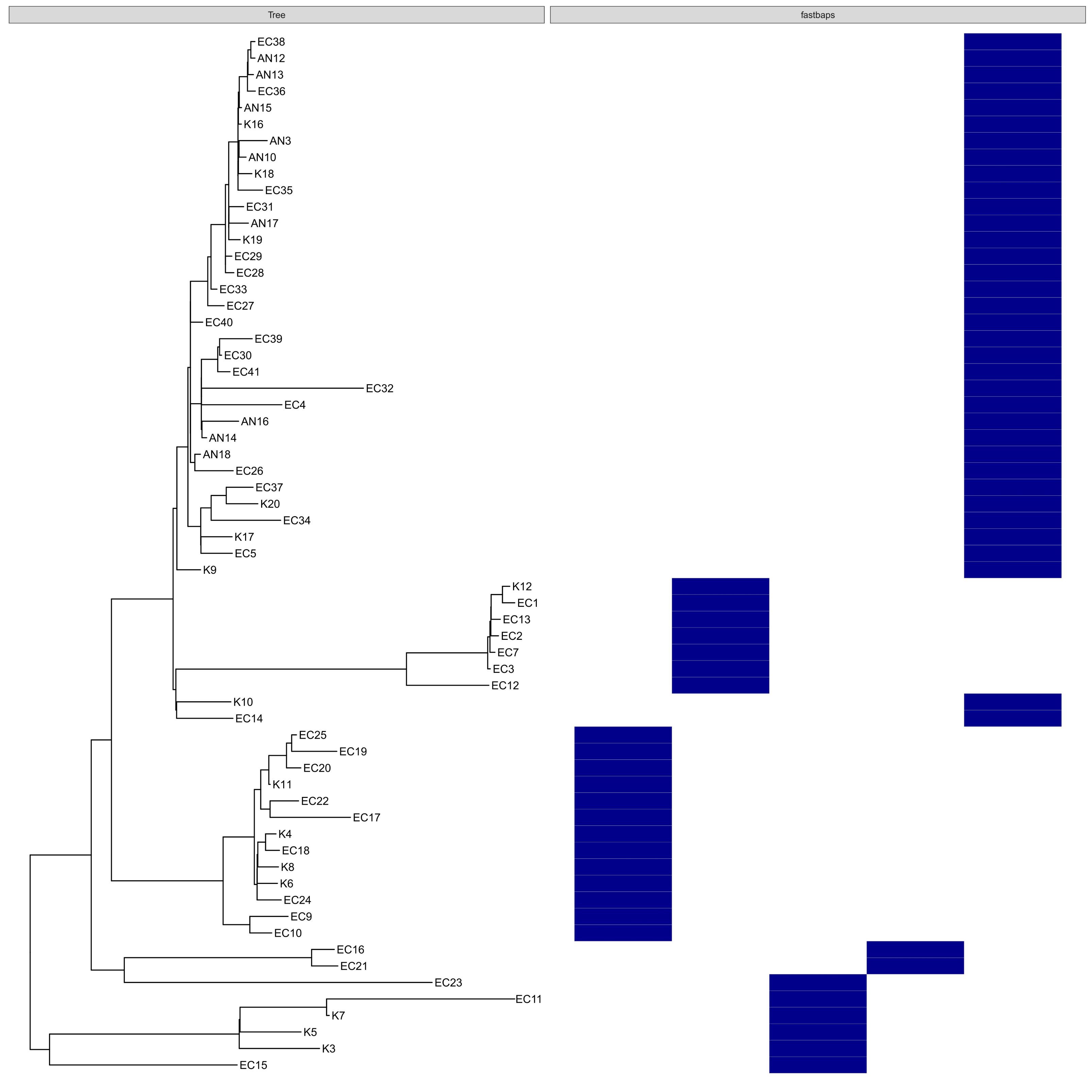

Supplement: Supplementary file 1 [file antibiotics-13-00363-s001.zip › Supplementary Figure S2.JPEG]
